# Supplementary material for: Village dogs match pet dogs in reading human facial expressions
Source: PeerJ. 2023 Jul 6;11:e15601. doi: 10.7717/peerj.15601 (PMC10329818; doi:10.7717/peerj.15601)
Supplement: Supplemental Information 1 — Additional information on statistics [file peerj-11-15601-s001.docx]

**Supplementary material.**

**Table**

Following are reported for all the models; the estimates, together with standard errors, tests, confidence limits, as well as minimum and maximum estimates derived after excluding individuals one at a time.

Table 1a_Proximity. ^a^ Dummy coded with free-ranging dog being the reference category; ^b^ Dummy coded with condition angry as the reference category; ^c^ Dummy coded with female as the reference category; ^d^ Dummy coded with body condition normal as the reference category; ^e^ Not indicated because having a limited interpretation; ^f^ The indicated likelihood ratio test refers to the overall effect of the respective interaction (tested by comparing the full model with a corresponding reduced model lacking the interaction).

| **Term** | **Estimate** | **SE** | ***z* or** *χ^2^* | ***P*** | **Lower Cl** | **Upper Cl** | **Min** | **Max** |
| --- | --- | --- | --- | --- | --- | --- | --- | --- |
| Intercept | -0.148 | 0.245 |  | NI^e^ | -0.639 | 0.321 | -0.306 | 0.210 |
| Group(PdA)^a^ | 1.013 | 0.348 |  | NI^e^ | 0.346 | 1.739 | 0.612 | 1.299 |
| Group(PdG)^a^ | 0.879 | 0.362 |  | NI^e^ | 0.132 | 1.600 | 0.481 | 1.142 |
| Condition(happy)^b^ | -0.683 | 0.335 |  | NI^e^ | -1.440 | -0.002 | -1.207 | -0.458 |
| Condition(neutral)^b^ | -0.285 | 0.325 |  | NI^e^ | -0.924 | 0.360 | -0.774 | -0.010 |
| Sex(m)^c^ | 0.048 | 0.173 | 0.280 | 0.780 | -0.273 | 0.427 | -0.043 | 0.127 |
| Body condition (thin)^d^ | 0.867 | 0.329 | 2.635 | 0.008 | 0.236 | 1.507 | 0.646 | 1.100 |
| Group(PdA)*Condition(happy) | 0.695 | 0.487 | 5.722 | 0.221^f^ | -0.258 | 1.729 | 0.123 | 1.337 |
| Group(PdA)*Condition(neutral) | 0.127 | 0.479 |  |  | -0.866 | 1.065 | -0.177 | 0.595 |
| Group(PdG)*Condition(happy) | 1.190 | 0.505 |  |  | 0.236 | 2.307 | 0.806 | 1.837 |
| Group(PdG)*Condition(neutral) | 0.155 | 0.506 |  |  | -0.840 | 1.187 | -0.859 | 0.877 |

Table 1b_Proximity. Results of the model lacking the interaction between group and condition (the full-null model comparison between the initial model including the interaction- see Table 1a- and a reduced model lacking the predictors group, condition and their interaction was significant: *χ^2^*=36.88, *df*=8, *P*<0.001 ). ^a^ Dummy coded with free-ranging dog as the reference category; ^b^ Dummy coded with condition angry as the reference category; ^c^ Dummy coded with female as the reference category; ^d^ Dummy coded with body condition normal as the reference category. The difference between PdA and PdG was estimated as 0.073±0.214, *z*=0.341, *P*=0.733. The difference between happy and neutral was estimated as 0.100±0.204, *z*=-0.490, *P*=0.624.

| **Term** | **Estimate** | **SE** | ***z*** | ***P*** |
| --- | --- | --- | --- | --- |
| Intercept | -0.316 | 0.209 | -1.514 | 0.130 |
| Group(PdA)^a^ | 1.262 | 0.219 | 5.770 | <0.001 |
| Group(PdG)^a^ | 1.323 | 0.228 | 5.808 | <0.001 |
| Condition(happy)^b^ | -0.223 | 0.284 | -0.784 | 0.433 |
| Condition(neutral) ^b^ | -0.199 | 0.205 | -0.971 | 0.332 |
| Sex(m)^c^ | 0.049 | 0.168 | 0.289 | 0.773 |
| Body condition (thin)^d^ | 0.836 | 0.345 | 2.425 | 0.015 |

Table 2_Eating all food. ^a^ Dummy coded with free-ranging dog being the reference category; ^b^ Dummy coded with condition angry as the reference category; ^c^ Dummy coded with female as the reference category; ^d^ Dummy coded with body condition normal as the reference category; ^e^ Not indicated because having a limited interpretation; ^f^ The indicated likelihood ratio test refers to the overall effect of the respective interaction (tested by comparing the full model with a corresponding reduced model lacking the interaction).

| **Term** | **Estimate** | **SE** | ***z* or** *χ^2^* | ***P*** | **Lower Cl** | **Upper Cl** | **Min** | **Max** |
| --- | --- | --- | --- | --- | --- | --- | --- | --- |
| Intercept | -0.162 | 0.439 |  | NI^e^ | -1.126 | 0.718 | -0.737 | 0.691 |
| Group(PdA)^a^ | 0.759 | 0.637 |  | NI^e^ | -0.515 | 2.288 | 0.035 | 1.348 |
| Group(PdG)^a^ | 2.226 | 0.865 |  | NI^e^ | 0.891 | 11.548 | 1.394 | 18.810 |
| Condition(happy)^b^ | -0.601 | 0.614 |  | NI^e^ | -1.993 | 0.650 | -1.592 | 0.069 |
| Condition(neutral)^b^ | -1.186 | 0.629 |  | NI^e^ | -2.774 | -0.073 | -1.542 | -0.637 |
| Sex(m)^c^ | 0.145 | 0.359 | 0.404 | 0.686 | -0.587 | 0.957 | -0.050 | 0.587 |
| Body condition (thin)^d^ | 0.696 | 0.601 | 1.158 | 0.247 | -0.673 | 2.358 | 0.111 | 0.942 |
| Group(PdA)*Condition(happy) | 2.189 | 1.076 | 11.948 | 0.018 ^f^ | 0.214 | 12.852 | 1.514 | 3.141 |
| Group(PdA)*Condition(neutral) | 2.793 | 1.092 |  |  | 0.816 | 13.398 | 2.237 | 3.716 |
| Group(PdG)*Condition(happy) | -0.586 | 1.107 |  |  | -10.131 | 1.803 | -1.248 | 0.638 |
| Group(PdG)*Condition(neutral) | -0.829 | 1.095 |  |  | -10.217 | 1.351 | -18.187 | -0.052 |

Table 3a_Tail wagging. ^a^ Dummy coded with free-ranging dog as the reference category; ^b^ Dummy coded with condition angry as the reference category; ^c^ Dummy coded with female as the reference category; ^d^ Dummy coded with body condition normal as the reference category; ^e^ Not indicated because having a limited interpretation; ^f^ The indicated likelihood ratio test refers to the overall effect of the respective interaction (tested by comparing the full model with a corresponding reduced model lacking the interaction).

| **Term** | **Estimate** | **SE** | ***z* or** *χ^2^* | ***P*** | **Lower Cl** | **Upper Cl** | **Min** | **Max** |
| --- | --- | --- | --- | --- | --- | --- | --- | --- |
| Intercept | -0.610 | 0.229 |  | NI^e^ | -1.078 | -0.131 | -0.717 | -0.518 |
| Group(PdA)^a^ | -1.066 | 0.336 |  | NI^e^ | -1.706 | -0.478 | -1.241 | -0.789 |
| Group(PdG)^a^ | -0.742 | 0.355 |  | NI^e^ | -1.458 | -0.075 | -1.038 | -0.630 |
| Condition(happy)^b^ | -0.494 | 0.309 |  | NI^e^ | -1.143 | 0.159 | -0.555 | -0.431 |
| Condition(neutral)^b^ | -0.044 | 0.324 |  | NI^e^ | -0.697 | 0.584 | -0.326 | 0.150 |
| Sex(m)^c^ | -0.059 | 0.165 | -0.356 | 0.722 | -0.388 | 0.281 | -0.275 | 0.031 |
| Body condition (thin)^d^ | 0.280 | 0.302 | 0.926 | 0.354 | -0.313 | 0.876 | 0.158 | 0.380 |
| Group(PdA)*Condition(happy) | 1.029 | 0.453 | 6.133 | 0.189^f^ | 0.129 | 1.935 | 0.792 | 1.113 |
| Group(PdA)*Condition(neutral) | 0.330 | 0.505 |  |  | -0.652 | 1.325 | -0.536 | 0.708 |
| Group(PdG)*Condition(happy) | 0.242 | 0.465 |  |  | -0.740 | 1.202 | 0.116 | 0.448 |
| Group(PdG)*Condition(neutral) | -0.357 | 0.608 |  |  | -1.400 | 0.602 | -0.591 | 0.002 |

Table 3b_Tail wagging. Results of the model lacking the interaction between group and condition (the full-null model comparison between the initial model including the interaction- see Table 2a- and a reduced model lacking the predictors group, condition and their interaction was significant: *χ^2^*=21.1, *df*=8, *P*=0.006). ^a^ Dummy coded with free-ranging dog as the reference category; ^b^ Dummy coded with condition angry as the reference category; ^c^ Dummy coded with female as the reference category; ^d^ Dummy coded with body condition normal as the reference category. The difference between PdA and PdG was estimated as 0.152±0.199, *z*=-0.765, *P*=0.444. The difference between happy and neutral was estimated as 0.076±0.192, *z*=-0.397, *P*=0.691.

| **Term** | **Estimate** | **SE** | ***z*** | ***P*** |
| --- | --- | --- | --- | --- |
| Intercept | -0.701 | 0.189 | -3.715 | <0.001 |
| Group(PdA)^a^ | -0.642 | 0.194 | -3.302 | <0.001 |
| Group(PdG)^a^ | -0.794 | 0.204 | -3.896 | <0.001 |
| Condition(happy)^b^ | -0.067 | 0.191 | -0.351 | 0.725 |
| Condition(neutral) ^b^ | 0.009 | 0.187 | 0.049 | 0.960 |
| Sex(m)^c^ | -0.151 | 0.158 | -0.958 | 0.338 |
| Body condition (thin)^d^ | 0.208 | 0.295 | 0.704 | 0.481 |

Table 4a_Gaze aversion. ^a^ Dummy coded with free-ranging dog as the reference category; ^b^ Dummy coded with condition angry as the reference category; ^c^ Dummy coded with female as the reference category; ^d^ Dummy coded with body condition normal as the reference category; ^e^ Not indicated because having a limited interpretation; ^f^ The indicated likelihood ratio test refers to the overall effect of the respective interaction (tested by comparing the full model with a corresponding reduced model lacking the interaction).

| **Term** | **Estimate** | **SE** | ***z* or** *χ^2^* | ***P*** | **Lower Cl** | **Upper Cl** | **Min** | **Max** |
| --- | --- | --- | --- | --- | --- | --- | --- | --- |
| Intercept | -3.652 | 0.296 |  | NI^e^ | -4.321 | -3.161 | -3.980 | -3.404 |
| Group(PdA)^a^ | -1.849 | 0.451 |  | NI^e^ | -2.876 | -0.990 | -2.199 | -1.527 |
| Group(PdG)^a^ | -0.800 | 0.419 |  | NI^e^ | -1.676 | 0.092 | -1.146 | -0.469 |
| Condition(happy)^b^ | -1.546 | 0.443 |  | NI^e^ | -2.643 | -0.716 | -1.928 | -0.996 |
| Condition(neutral)^b^ | -0.814 | 0.393 |  | NI^e^ | -1.641 | -0.054 | -0.925 | -0.732 |
| Sex(m)^c^ | 0.049 | 0.223 | 0.219 | 0.827 | -0.446 | 0.489 | -0.129 | 0.148 |
| Body condition (thin)^d^ | -0.312 | 0.481 | -0.650 | 0.516 | -1.506 | 0.480 | -0.923 | -0.020 |
| Group(PdA)*Condition(happy) | 1.114 | 0.690 | 5.500 | 0.240^f^ | -0.339 | 2.684 | 0.563 | 1.742 |
| Group(PdA)*Condition(neutral) | 0.834 | 0.631 |  |  | -0.486 | 2.201 | 0.140 | 1.173 |
| Group(PdG)*Condition(happy) | 1.234 | 0.625 |  |  | -0.029 | 2.658 | 0.678 | 1.457 |
| Group(PdG)*Condition(neutral) | 0.350 | 0.606 |  |  | -0.957 | 1.669 | -0.082 | 0.530 |

Table 4b_Gaze aversion. Results of the model lacking the interaction between group and condition (the full-null model comparison between the initial model including the interaction- see Table 4a- and a reduced model lacking the predictors group, condition and their interaction was highly significant: *χ^2^*=30.306, *df*=8, *P*<0.001 ). ^a^ Dummy coded with free-ranging dog as the reference category; ^b^ Dummy coded with condition angry as the reference category; ^c^ Dummy coded with female as the reference category-, ^d^ Dummy coded with body condition normal as the reference category. The difference between PdA and PdG was estimated as 0.943±0.288, *z*=3.269, *P*=0.001. The difference between happy and neutral was estimated as 0.309±0.287, *z*= 1.081, *P*=0.279.

| **Term** | **Estimate** | **SE** | ***z*** | ***P*** |
| --- | --- | --- | --- | --- |
| Intercept | -3.934 | 0.245 | -16.031 | <0.001 |
| Group(PdA)^a^ | -1.266 | 0.287 | -4.404 | <0.001 |
| Group(PdG)^a^ | -0.323 | 0.272 | -1.185 | 0.236 |
| Condition(happy)^b^ | -0.809 | 0.274 | -2.956 | 0.003 |
| Condition(neutral) ^b^ | -0.499 | 0.261 | -1.909 | 0.056 |
| Sex(m)^c^ | 0.031 | 0.222 | 0.142 | 0.887 |
| Body condition (thin)^d^ | -0.282 | 0.483 | -0.585 | 0.558 |

Table 5a_Looking. ^a^ Dummy coded with free-ranging dog as the reference category; ^b^ Dummy coded with condition angry as the reference category; ^c^ Dummy coded with female as the reference category; ^d^ Dummy coded with body condition normal as the reference category; ^e^ Not indicated because having a limited interpretation; ^f^ The indicated likelihood ratio test refers to the overall effect of the respective interaction (tested by comparing the full model with a corresponding reduced model lacking the interaction).

| **Term** | **Estimate** | **SE** | ***z* or** *χ^2^* | ***P*** | **Lower Cl** | **Upper Cl** | **Min** | **Max** |
| --- | --- | --- | --- | --- | --- | --- | --- | --- |
| Intercept | -0.639 | 0.148 |  | NI^e^ | -0.944 | -0.367 | -0.665 | -0.589 |
| Group(PdA)^a^ | -0.589 | 0.221 |  | NI^e^ | -1.039 | -0.157 | -0.636 | -0.476 |
| Group(PdG)^a^ | -0.052 | 0.218 |  | NI^e^ | -0.488 | 0.352 | -0.363 | 0.046 |
| Condition(happy)^b^ | -0.493 | 0.210 |  | NI^e^ | -0.888 | -0.116 | -0.653 | -0.322 |
| Condition(neutral)^b^ | -0.096 | 0.196 |  | NI^e^ | -0.489 | 0.272 | -0.196 | 0.002 |
| Sex(m)^c^ | -0.002 | 0.110 | -0.015 | 0.988 | -0.215 | 0.225 | -0.091 | 0.054 |
| Body condition (thin)^d^ | 0.249 | 0.200 | 1.28 | 0.212 | -0.138 | 0.643 | 0.047 | 0.484 |
| Group(PdA)*Condition(happy) | 0.653 | 0.312 | 5.580 | 0.233^f^ | 0.076 | 1.270 | 0.496 | 0.813 |
| Group(PdA)*Condition(neutral) | 0.059 | 0.307 |  |  | -0.555 | 0.692 | -0.096 | 0.183 |
| Group(PdG)*Condition(happy) | 0.608 | 0.310 |  |  | 0.034 | 1.225 | 0.413 | 0.781 |
| Group(PdG)*Condition(neutral) | 0.062 | 0.307 |  |  | -0.579 | 0.676 | -0.036 | 0.193 |

Table 5b_Looking. Results of the model lacking the interaction between group and condition (the full-null model comparison between the initial model including the interaction- see Table 3a- and a reduced model lacking the predictors group, condition and their interaction was significant: *χ^2^*=22.349, *df*=8, *P*= 0.004). ^a^ Dummy coded with free-ranging dog as the reference category; ^b^ Dummy coded with condition angry as the reference category; ^c^ Dummy coded with female as the reference category;  ^d^ Dummy coded with body condition normal as the reference category. The difference between PdA and PdG was estimated as 0.521±0.135, *z*= 3.837, *P*<0.001. The difference between happy and neutral was estimated as 0.045± 0.131, *z*= 0.351, *P*=0.725.

| **Term** | **Estimate** | **SE** | ***z*** | ***P*** |
| --- | --- | --- | --- | --- |
| Intercept | -0.734 | 0.131 | -5.605 | <0.001 |
| Group(PdA)^a^ | -0.377 | 0.134 | -2.813 | 0.005 |
| Group(PdG)^a^ | 0.150 | 0.134 | 1.115 | 0.265 |
| Condition(happy)^b^ | -0.165 | 0.181 | -0.915 | 0.360 |
| Condition(neutral) ^b^ | -0.061 | 0.126 | -0.482 | 0.630 |
| Sex(m)^c^ | -0.005 | 0.107 | -0.048 | 0.962 |
| Body condition (thin)^d^ | 0.177 | 0.207 | 0.857 | 0.391 |

Table 6. Vif values for all the models.

| **Models** | **VIF values** | | | |
| --- | --- | --- | --- | --- |
|  | Group | Condition | Sex | Body condition |
| All | 1.091877 | 1.013654 | 1.019370 | 1.195636 |

**Table 7. Description of the models.**

| **Model** | **Full model** | **Null model** |
| --- | --- | --- |
| Proximity | Proximity(prop) ~group*condition+sex+body condition+(random effects) | Proximity(prop) ~1+sex+body condition+(random effects) |
| Eat available food | Eat(yes or no) ~group*condition+sex+body condition+(random effects) | Eat(yes or no) ~1+sex+body condition+(random effects) |
| Tail wagging | Tail wagging(prop) ~group*condition+sex+body condition+(random effects) | Proximity(prop) ~1+sex+body condition+(random effects) |
| Gaze aversion | Aversive gazes ~group*condition+sex+body condition+offset+(random effects) | Looking(prop) ~1+sex+body condition+offset+ (random effects) |
| Looking at the experimenter | Looking(prop) ~group*condition+sex+body condition+(random effects) | Proximity(prop) ~1+sex+body condition+(random effects) |
